# Differential effects of Mendelian *GDAP1* clinical variants on mitochondria-lysosome membrane contacts sites

**DOI:** 10.1242/bio.059707

**Published:** 2023-04-03

**Authors:** Lara Cantarero, Gisela García-Vargas, Janet Hoenicka, Francesc Palau

**Affiliations:** ^1^ Laboratory of Neurogenetics and Molecular Medicine – IPER, Institut de Recerca Sant Joan de Déu, 08950, Barcelona, Spain; ^2^ Centro de Investigación Biomédica en Red de Enfermedades Raras (CIBERER), ISCIII, 08950, Barcelona, Spain; ^3^ Department of Genetic Medicine – IPER, Hospital Sant Joan de Déu, 08950, Barcelona, Spain; ^4^ Division of Pediatrics, Faculty of Medicine and Health Sciences, University of Barcelona, 08036, Barcelona, Spain; ^5^ ERN-ITHACA

**Keywords:** Charcot-Marie-Tooth disease, GDAP1, Lysosome, Membrane contact sites, Mitochondria

## Abstract

*GDAP1* pathogenic variants cause Charcot-Marie-Tooth (CMT) disease, the most common hereditary motor and sensory neuropathy. CMT-*GDAP1* can be axonal or demyelinating, with autosomal dominant or recessive inheritance, leading to phenotypic heterogeneity. Recessive *GDAP1* variants cause a severe phenotype, whereas dominant variants are associated with a milder disease course. GDAP1 is an outer mitochondrial membrane protein involved in mitochondrial membrane contact sites (MCSs) with the plasmatic membrane, the endoplasmic reticulum (ER), and lysosomes. In *GDAP1*-deficient models, the pathophysiology includes morphological defects in mitochondrial network and ER, impaired Ca^2+^ homeostasis, oxidative stress, and mitochondrial MCSs defects. Nevertheless, the underlying pathophysiology of dominant variants is less understood.

Here, we study the effect upon mitochondria–lysosome MCSs of two *GDAP1* clinical variants located in the α-loop interaction domain of the protein. p.Thr157Pro dominant variant causes the increase in these MCSs that correlates with a hyper-fissioned mitochondrial network. In contrast, p.Arg161His recessive variant, which is predicted to significantly change the contact surface of GDAP1, causes decreased contacts with more elongated mitochondria.

Given that mitochondria–lysosome MCSs regulate Ca^2+^ transfer from the lysosome to mitochondria, our results support that *GDAP1* clinical variants have different consequences for Ca^2+^ handling and that could be primary insults determining differences in severity between dominant and recessive forms of the disease.

## Teaser

**Summary:** The relevance of mitochondria-lysosome membrane contact sites dysfunction as a primary event in axonopathies etiology, and as a key signaling hub opening novel therapeutic strategies in peripheral neuropathies, especially in Charcot-Marie-Tooth disease.

## INTRODUCTION

Pathogenic variants in the *GDAP1* gene cause Charcot-Marie-Tooth (CMT) disease, a motor and sensory neuropathy, which is the most common hereditary neuromuscular disorder. CMT-*GDAP1* can be expressed as an autosomal recessive demyelinating neuropathy (Baxter et al., 2002) or as an axonopathy, either recessive (Cuesta et al., 2002) or dominant (Claramunt et al., 2005; Sivera et al., 2010; Zimon et al., 2011). *GDAP1* recessive pathogenic variants are associated with early-onset and severe disease, with most patients being wheelchair-bound in their second decade and having vocal cord paresis (Sevilla et al., 2008). It has been suggested that recessive variants that result in truncated proteins cause a more severe phenotype, while missense variants may be associated with a slightly milder course (Cassereau et al., 2011). However, *GDAP1* dominant pathogenic variants used to be associated with a much milder disease course (Sivera et al., 2017), characterized by adult-onset, predominantly distal involvement, and slow progression (Sivera et al., 2010; Zimon et al., 2011). This clinical and Mendelian heterogeneity of CMT-*GDAP1* could be associated with differences in the underlying pathophysiological processes (Pla-Martin et al., 2013).

*GDAP1* encodes an integral protein of the outer mitochondrial membrane (OMM) (Niemann et al., 2005; Pedrola et al., 2005), which is an atypical glutathione S-transferase (GST) (Marco et al., 2004; Nguyen et al., 2021) with membrane-remodeling activity (Huber et al., 2016). The GDAP1 protein has two GST domains separated by the α-loop substrate-binding domain necessary for GDAP1-protein interactions (Googins et al., 2020; Marco et al., 2004; Nguyen et al., 2021), a hydrophobic domain (HD) and a C-terminal transmembrane domain responsible for its anchoring to the OMM.

GDAP1 participates in the dynamics of the mitochondrial network, specifically in fission processes (Niemann et al., 2005; Pedrola et al., 2008; Pijuan et al., 2022; Pla-Martin et al., 2013), and in the membrane contact sites (MCSs) between mitochondria and other organelles (Cantarero et al., 2021; Pla-Martin et al., 2013). When GDAP1 is depleted, cells exhibit structural defects in the mitochondrial network and endoplasmic reticulum (ER) cisternae and they presented reduced mitochondrial-ER MCSs known as mitochondria-associated membranes (MAM) (Pla-Martin et al., 2013). GDAP1-associated MAMs defects alter calcium homeostasis (Barneo-Munoz et al., 2015; Civera-Tregon et al., 2021; Gonzalez-Sanchez et al., 2017; Pla-Martin et al., 2013).

Recently, we described that GDAP1 is located at mitochondria–lysosome MCSs (Cantarero et al., 2021), which have been identified as mitochondrial fission regulators via the lysosomal GTPase Ras-related Protein Rab-7 (RAB7) (Wong et al., 2018). In mitochondria–lysosome MCSs, GDAP1 interacts with the lysosome-associated membrane protein-1 (LAMP-1) (Cantarero et al., 2021). Moreover, GDAP1 depletion decreased the number and duration of mitochondria–lysosome contacts, affecting mitochondrial and lysosomal morphology, and impairing basal autophagy (Cantarero et al., 2021). The effect of *GDAP1* dominant variants on the biology of mitochondria-lysosome MCSs is not known.

Here we aimed to study the effect of dominant and recessive *GDAP1* missense pathogenic variants located inside the α-loop domain on the structure and function of mitochondria-lysosome MCSs. We found that *GDAP1* recessive and dominant variants have different effects on these mitochondrial MCSs.

## RESULTS AND DISCUSSION

### Recessive and dominant *GDAP1* pathogenic variants have differential effects on mitochondria-lysosome MCSs

For this study, we selected the pathogenic *GDAP1* missense variants p.Arg161His (recessive) (Ammar et al., 2003) and p.Thr157Pro (dominant) (Claramunt et al., 2005), which lie within the GDAP1 α-loop interaction domain and the CMT-related mutation cluster (Googins et al., 2020; Marco et al., 2004; Pla-Martin et al., 2013). Additionally, p.Arg161His and p.Thr157Pro are fully conserved among phylogenetically distant vertebrate species.

**Fig. 1.**
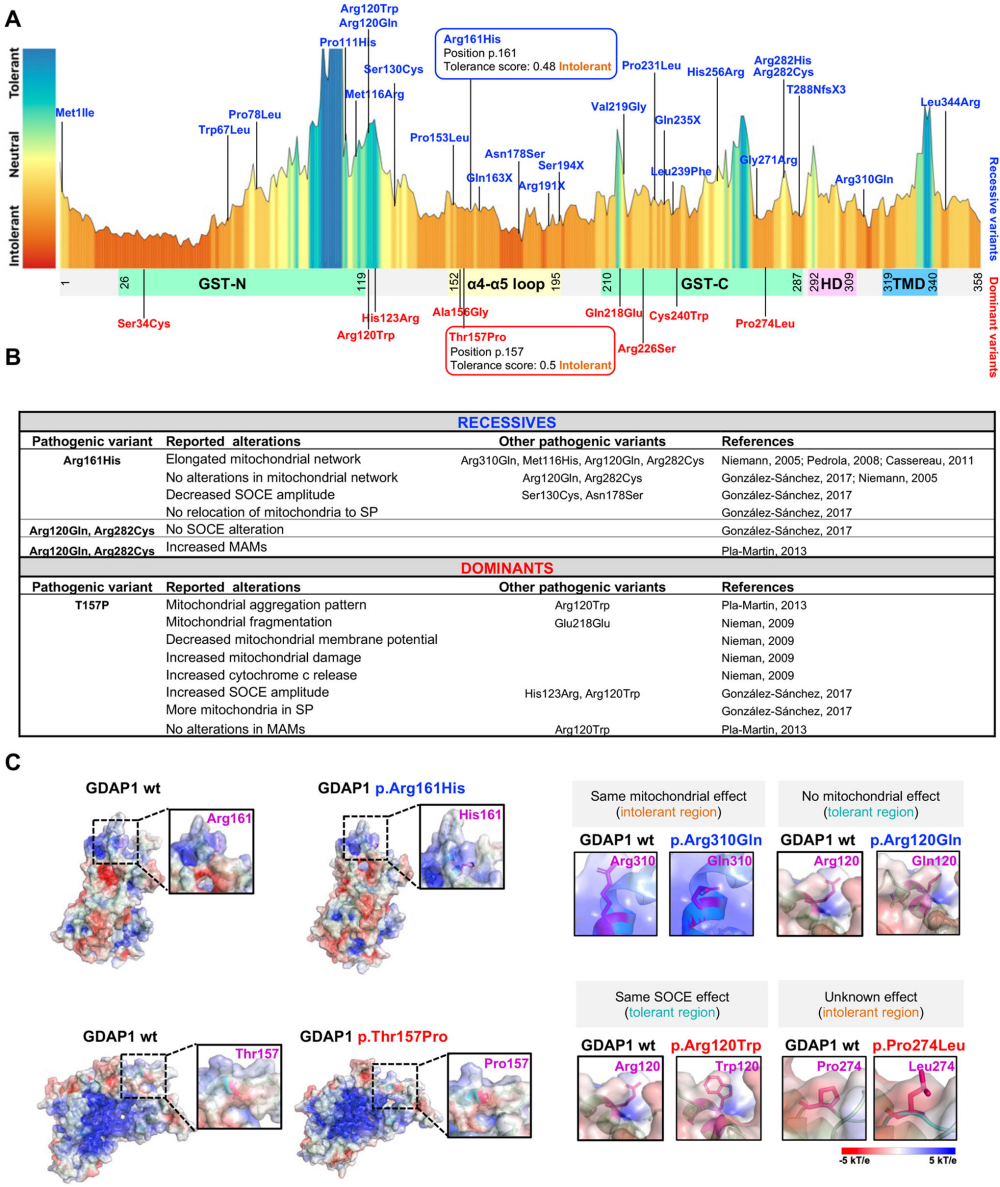
***In-silico* biology study of *GDAP1* recessive and dominant clinical variants.** (A) An intolerance landscape plot generated by MetaDome for recessive (blue) and dominant (red) *GDAP1* clinical variants (top panel) and a schematic outline of GDAP1 protein showing its domains (lower panel). The two *GDAP1* pathogenic variants used in this study are framed. (B) Summary of reported cellular alterations caused by *GDAP1* recessive and dominant clinical variants. (C) Electrostatic surface potential of GDAP1 with the residues of interest labeled in magenta. Upper panel: GDAP1 wild type and recessive clinical variants p.Arg161His (this work), p.Arg131Gln and p.Arg120Gln. Lower panel: GDAP1 wild type and dominant clinical variants p.Thr157Pro (this work), p.Arg120Trp and p.Pro274Leu. GST: glutathione-S transferase; HD: hydrophobic domain; TM: transmembrane domain; SOCE: Store-operated calcium entry; SP: subplasmalemmal fraction; MAMs: mitochondria-associated membranes.

We first evaluated p.Arg161His and p.Thr157Pro pathogenicity using different *in-silico* predictors tools and data mining. The Mutation Taster (Schwarz et al., 2014) classified both variants as disease-causing and the Combined Annotation Dependent Depletion (CADD) (Rentzsch et al., 2019), a pathogenicity predictor that integrates multiple algorithms, scored them above the pathogenic threshold (32 for p.Arg161His and 23.2 for p.Thr157Pro). Furthermore, according to the American College of Medical Genetics and Genomics (ACMG) guidelines (Richards et al., 2015), these variants were classified as pathogenic (p.Arg161His) and likely pathogenic (p.Thr157Pro). We also investigated the tolerance domain landscape using MetaDome (Wiel et al., 2019), which showed that both p.Arg161His and p.Thr157Pro reside in change-intolerant positions of GDAP1 (Fig. 1A).

We further extended the study using MetaDome with additional *GDAP1* clinical variants. The MetaDome profile showed that both recessive and dominant variants are distributed throughout the entire GDAP1 protein, localizing in tolerant and intolerant regions, with no specificity in any domain (Fig. 1A).

Regarding to the effect of p.Arg161His and p.Thr157Pro on mitochondrial biology, these variants have been associated to defects in mitochondrial morphology and membrane contacts sites, as well as in calcium homeostasis (Fig. 1B). Other pathogenic *GDAP1* variants share some of these alterations at the cellular level, while some vary in their effects, showing great phenotypic heterogeneity. For example, the recessive variants p.Arg161His and p.Arg310Gln produce a more elongated and interconnected mitochondrial network, whereas the recessive variant p.Arg120Gln does not produce significant changes in mitochondrial network morphology. In contrast, the dominant variant p.Thr157Pro produces aggregated mitochondria or mitochondrial hyper fragmentation.

**Fig. 2.**
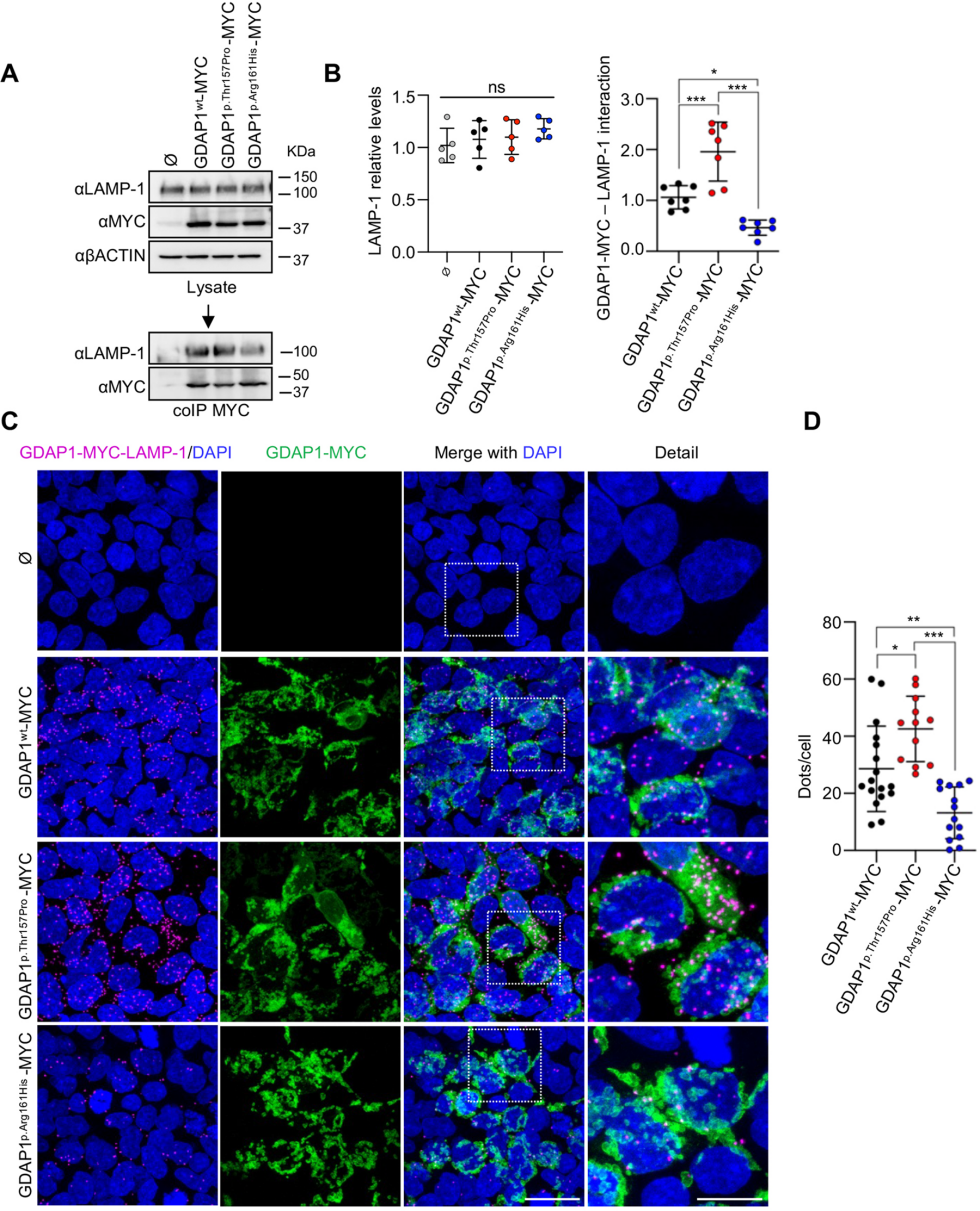
**Differential effects on mitochondria-lysosome MCSs by *GDAP1* pathogenic variants.** (A) Co-IP assay of GDAP1-MYC and endogenous LAMP-1 interaction in HEK293T cells transfected with the following constructs: pCMV-AC Ø, pCMV-GDAP1^wt^-MYC, pCMV-GDAP1^p.Thr157Pro^-MYC and pCMV-GDAP1^p.Arg161His^-MYC. (B) Left panel: quantification of LAMP-1 relative levels. Data represents mean±s.d. and individual values are displayed as dots. ANOVA followed by Tukey's *post hoc* test. Five independent experiments. *ns: not significant. Right panel: quantification of GDAP1-MYC and endogenous LAMP-1 interaction. Data represents mean±s.d. and individual values are displayed as dots. ANOVA followed by Tukey's *post hoc* test. Five independent experiments. *P<0.05, ***P<0.001. (C) Proximity ligation assay of GDAP1-MYC and endogenous LAMP-1 in HEK293T cells transfected with the following constructs: pCMV-AC Ø, pCMV-GDAP1^wt^-MYC, pCMV-GDAP1^p.Thr157Pro^-MYC and pCMV-GDAP1^p.Arg161His^-MYC. Complementary images with MYC (GDAP1) staining and magnification details are shown. Scale bar: 25 µm; detail 10 µm. (D) Number of dots per cell quantification. Data represent mean±s.d. and individual values are displayed as dots (*n*=17 GDAP1, *n*=12 p.Thr157Pro, *n*=14 p.Arg161His fields from three independent experiments). ANOVA followed by Tukey's *post hoc* test. *P<0.05, **P<0.01, ***P<0.001.

Since ultrastructural data have shown that CMT-*GDAP1* pathogenic variants could affect the folding and stability of the protein, and modify its interactions with other molecules (Googins et al., 2020), we evaluated whether both p.Arg161His and p.Thr157Pro could alter the interaction between GDAP1 and its proteins partners. For this, we modeled an electrostatic potential map of GDAP1 protein using PyMOL (Schrodinger, 2015) (Fig. 2C). We observed that the model of p.Arg161His recessive variant showed an arginine residue predicted to be more exposed to the surface, whereas histidine residue, which is also charged positive but is hydrophobic, was hidden within the GDAP1 molecule. The same effect on the mitochondrial network was found in the recessive variant p.Arg310Gln, whereas the p.Arg120Gln variant, which did not alter the mitochondrial morphology, did not show any structural change. On the contrary, we did not observe significant differences in the residues involved in the dominant pathogenic variants p.Thr157Pro, p.Arg120Trp and p.Pro274Leu. Indeed, CMT-*GDAP1* dominant variants are mostly surface residues with the potential to facilitate protein–protein interactions, while recessive variants can cause more buried residues (Googins et al., 2020). All of these *in-silico* analyses and data mining were consistent with our hypothesis of a differential effect of dominant and recessive *GDAP1* clinical variants on mitochondria–lysosome MCSs.

As GDAP1–LAMP-1 is the only known tether-pair of mitochondria–lysosome MCSs (Cantarero et al., 2021), here we investigated the effect of p.Arg161His and p.Thr157Pro on these contacts by measuring GDAP1–LAMP-1 interaction. HEK293T cells were transfected with constructs overexpressing GDAP1-MYC fusion proteins of wild-type, p.Thr157Pro and p.Arg161His variants. First, we studied by co-immunoprecipitation (co-IP) whether these variants affect the interaction between GDAP1 and LAMP-1 (Fig. 2A). In agreement with PyMOL findings, co-IP experiments showed a significant increase in binding for GDAP1^p.Thr157Pro^-MYC (dominant), while GDAP1^p.Arg161His^-MYC (recessive) showed a significant reduction (Fig. 2A-B). We then quantified GDAP1–LAMP-1 interaction by proximity ligation assay (PLA) (Fig. 2C). Consistent with co-IP results, GDAP1^p.Arg161His^-MYC showed significantly less interaction with LAMP-1 (Fig. 2D). These results are in line with the reduction of GDAP1–LAMP-1 interaction observed in fibroblasts from a CMT patient who is a homozygous carrier of the recessive variant p.Trp67Leu located at the GST N-terminal domain (Pijuan et al., 2022). Therefore, *GDAP1* recessive variant inside or outside the α-loop domain decrease GDAP1–LAMP-1 interaction thus causing the loss of contact between mitochondria and lysosomes. In contrast, we observed a higher interaction between GDAP1^p.Thr157Pro^-MYC and LAMP-1 (Fig. 2D).

A differential effect of dominant and recessive *GDAP1* variants on intracellular calcium homeostasis has been reported (Gonzalez-Sanchez et al., 2017; Pla-Martin et al., 2013), specifically on store-operated calcium entry (SOCE). *GDAP1* dominant variants increase SOCE activity compared to wild type, whereas *GDAP1* recessive variants located inside the α-loop show a reduction of SOCE activity. Strikingly, those recessive variants located outside the α-loop domain did not affect SOCE activity, which may act by another mechanism. Thus, *GDAP1* recessive variants in the α-loop affect both SOCE activity and mitochondria–lysosome MCSs, while recessive variants outside the α-loop exclusively affect these contacts suggesting that the N-terminal GST domain is involved in the structural function of GDAP1 at mitochondria–lysosome MCSs. In addition, these contacts regulate mitochondrial calcium homeostasis through the lysosomal calcium efflux channel (transient potential mucolipin receptor 1, TRPML1) (Peng et al., 2020). In the mucolipidosis type IV (MLIV) recessive disease, caused by *TRMPL1* variants, there is an increased number and duration of mitochondria–lysosome contacts, with defective contact-dependent mitochondrial calcium uptake upon TRPML1 activation. It has been proposed that prolonged mitochondria–lysosome contacts in MLIV compensate for reduced calcium transfer due to the loss of function of the TRPML1 channel. In this work, the effects of the *GDAP1* recessive variant on mitochondria–lysosome MCSs can be explained by defects in its structural function in the contact, which would also reduce calcium transfer.

### Morphological defects in the mitochondrial network caused by *GDAP1* pathogenic variants

**Fig. 3.**
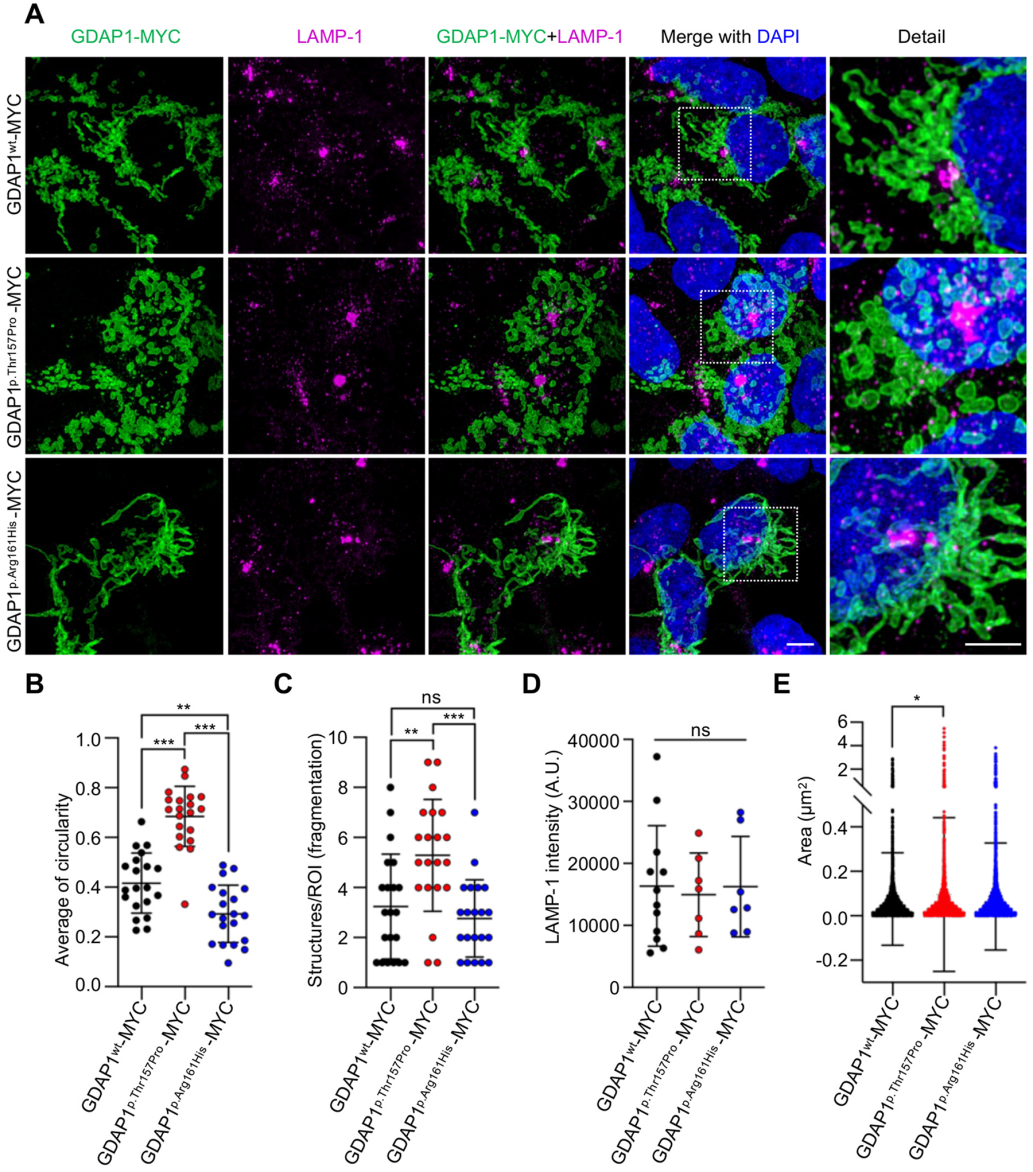
**Morphological defects in the mitochondrial network caused by *GDAP1* pathogenic variants.** (A) Representative images of GDAP1-MYC (green), endogenous LAMP-1 (magenta) and DAPI (blue) in HEK293T cells transfected with pCMV-GDAP1^wt^-MYC, pCMV-GDAP1^p.Thr157Pro^-MYC (dominant variant) and pCMV-GDAP1^p.Arg161His^-MYC (recessive variant). A magnification detail is shown. Scale bar: 5 µm. (B) Average of mitochondrial circularity quantification. Data represents mean±s.d. and individual values are displayed as dots (*n*=20 GDAP1, *n*=20 p.Thr157Pro, *n*=20 p.Arg161His ROIs from three independent experiments). ANOVA followed by Tukey's *post hoc* test. ***P<0.001. (C) Quantification of the number of mitochondrial structures per ROI (index of fragmentation). Data represents mean±s.d. and individual values are displayed as dots (*n*=21 GDAP1, *n*=21 p.Thr157Pro, *n*=21 p.Arg161His ROIs from three independent experiments). ANOVA followed by Tukey's *post hoc* test. **P<0.01, ***P<0.001. (D) LAMP-1 intensity quantification. Data represent mean±s.d. and individual values are displayed as dots (*n*=12 GDAP1, *n*=7 p.Thr157Pro, *n*=7 p.Arg161His fields from three independent experiments). ANOVA followed by Tukey's *post hoc* test. ns, not significant. A.U., arbitrary units. (E) Lysosome area quantification. Data represent mean±s.d. and individual values are displayed as dots (*n*=2763 GDAP1, *n*=2058 p.Thr157Pro, *n*=3273 p.Arg161His lysosomes from three independent experiments). ANOVA followed by Tukey's *post hoc* test. *P<0.05.

Since mitochondria–lysosome MCSs regulate mitochondrial fission (Wong et al., 2018) and *GDAP1* deficient cell models present abnormalities in the mitochondrial network and lysosomes (Cantarero et al., 2021), here we analyzed the effect of the two *GDAP1* variants in mitochondria and lysosome biology in transfected HEK293T cells (Fig. 3A). We observed a strong phenotype caused by *GDAP1* dominant variant p.Thr157Pro. In these cells, mitochondria presented a circular morphology (Fig. 3B) and the mitochondrial network was hyper-fissioned (Fig. 3C). These abnormalities could be related to mitochondria–lysosome MCSs increment caused by p.Thr157Pro enhancing mitochondrial fission. This scenario would cause defects in mitochondrial dynamics, which consequently may contribute to downstream phenotypes such as mitochondrial fragmentation. At this point, it is important to consider the experimental limitation of overexpressing pathogenic variants in a wild-type GDAP1 background and carefully infer gain-of-function phenotypes. The effect of p.Thr157Pro on the mitochondrial network is likely milder in a physiological setting; in contrast, the recessive *GDAP1* variant p.Arg161His caused more elongated mitochondria when compared with cells overexpressing wild-type GDAP1 (Fig. 3B), which is a previously described phenotype for this variant (Cassereau et al., 2011; Niemann et al., 2009; Pedrola et al., 2008). In fact, in fibroblasts from the patient carrying p.Trp67Leu *GDAP1* recessive variant, a similar mitochondrial morphology was described and categorized as a ‘tangled’ network because of diminished fission (Pijuan et al., 2022).

**Fig. 4.**
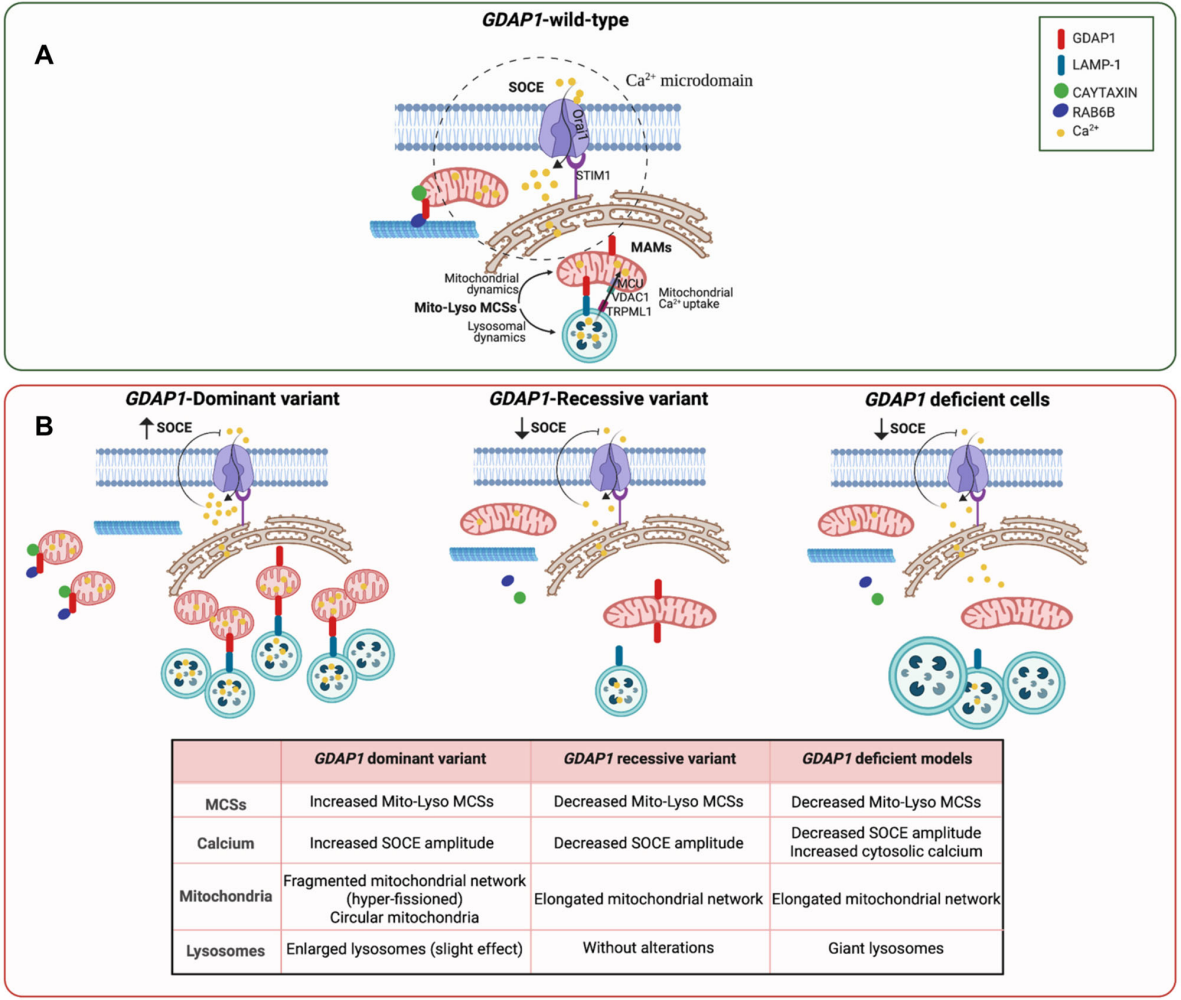
***GDAP1* pathogenic variants effect in mitochondria-lysosome MCSs biology.** (A) *GDAP1* wild-type: interaction between GDAP1 and the trafficking proteins Caytaxin and Rab6B allows mitochondria to approach Ca^2+^ microdomains between the ER and the plasma membrane after activation of storage-driven calcium entry (SOCE). This mitochondrial approach facilitates Ca^2+^ uptake and regulates ATP production by oxidative phosphorylation. Likewise, GDAP1 is located in MAMs and regulates mitochondria–lysosome MCSs (Mito-Lyso MCSs) through LAMP-1 interaction. These contacts allow (1) the bidirectional regulation of mitochondrial fission and lysosomal dynamics and (2) the calcium efflux from lysosomes to mitochondria through TRPML1, VDAC1 and MCU proteins. (B) *GDAP1*-pathogenic variants: in cells expressing dominant *GDAP1* variants (gain-of-function), mitochondria are already close to the plasma membrane under basal conditions by a dominant-negative mechanism, which could be related to increased SOCE activity affecting stimulation of mitochondrial respiration. In addition, mitochondria–lysosome MCSs are increased, probably also affecting Ca^2+^ homeostasis and causing hyperfission of the mitochondrial network. In contrast, recessive *GDAP1* variants (loss-of-function) and GDAP1 deficient cells (loss-of-function) cause impair mitochondrial movement and SOCE activity, and failure in stimulation of mitochondrial respiration. Mitochondria–lysosome MCSs are decreased as a consequence of less interaction with its partner LAMP-1, leading to an elongated and ‘tangled’ mitochondrial network. Finally, in GDAP1 deficient cells, giant lysosome with abnormal distribution are additionally observed.

Regarding lysosomes, the study of their morphology and distribution was performed by staining endogenous LAMP-1 in the transfected cells (Fig. 3A). We found no differences in the signal distribution and intensity of LAMP-1 (Fig. 3D), nor in LAMP-1 protein levels (Fig. 2B), which would indicate no effects on the number of lysosomes. Lysosomal area quantification showed a slight effect in cells overexpressing the dominant *GDAP1* variant p.Thr157Pro that presented a significant increase (Fig. 3E). Giant lysosomes have been reported in neural GDAP1 deficient models (Cantarero et al., 2021; Pijuan et al., 2022), however, our results after overexpressing *GDAP1* variants did not replicate these findings suggesting a cell lineage-dependent effect. Besides, we emphasize that *GDAP1* recessive variants, associated with a loss of function, are predicted to be unfunctional. Nevertheless, the overexpression in a *GDAP1* wild-type background might cover this lysosomal effect.

From a medical perspective, the differential effect of clinical pathogenic variants on mitochondria–lysosome MCSs, SOCE, and calcium homeostasis, seems to indicate that *GDAP1* dominant variants increase cellular and mitochondrial calcium levels while recessive loss-of-function variants cause a decrease. Given the importance of calcium in cellular homeostasis and mitochondria energy production, these differences highlight key mechanistic aspects of the pathophysiology of severe recessive forms of CMT-*GDAP1* compared with milder clinical dominant forms. Moreover, GDAP1 contains characteristic GST domains with special features and it has been proposed that it could regulate mitochondrial fission by acting as a cellular redox sensor, a process that, in turn, depends on cytosolic Ca^2+^ (Jeyaraju et al., 2009). With all these results, it is tempting to propose that GDAP1 could regulate the fission of the mitochondrial network and its connection with cellular physiology through calcium handling. Finally, the different cellular consequences of the pathogenic variants of *GDAP1* show the complex pathophysiology of Charcot-Marie-Tooth disease. This molecular and cellular knowledge is necessary for the design of differential and personalized therapies.

## MATERIALS AND METHODS

### Reagents

#### Plasmids

The following plasmids were used: pCMV6-AC-Ø, pCMV-GDAP1-MYC (wild-type), pCMV-GDAP1-MYC p.Thr157Pro (dominant variant), and pCMV-GDAP1-MYC p.Arg161His (recessive variant) (Gonzalez-Sanchez et al., 2017; Pla-Martin et al., 2013).

#### Antibodies

The following antibodies were used: β-ACTIN mouse monoclonal (Sigma-Aldrich, A5316; 1:8000 WB), LAMP-1 rabbit polyclonal (Abcam, ab24170, 1:500 IF), LAMP-1 mouse monoclonal (DSHB, H4A3; 1:500 WB), and MYC mouse monoclonal (Sigma-Aldrich, SAB2702192; 1:2000 WB; 1:200 IF).

### Bioinformatics and *in silico* analyses

The pathogenicity analysis of genetic variants was done with MetaDome (vemodeled.1) (Wiel et al., 2019). The electrostatic surfaces were modeled with APBS and PDB2PQR in the PyMOL Molecular Graphics System (Schrodinger, 2015). The classification of *GDAP1* variants was done using VarSome (last accessed & February 2023) according to the American College of Medical Genetics and Genomics (ACMG) standard guidelines. *In-silico* analyses of the genetic variants were performed using: MutationTaster (Schwarz et al., 2014) and CADD (Rentzsch et al., 2019).

### Cell culture

HEK293T cells were cultured in Dulbecco's Modified Eagles’ Medium high-glucose (Sigma-Aldrich, D5796) supplemented with 10% fetal bovine serum (Sigma-Aldrich, F7524), 2 mM L-glutamine (Sigma-Aldrich, G7513) and 100 mg/ml penicillin-streptomycin (Sigma-Aldrich, P4333), at 37°C in a 5% CO_2_ incubator. HEK293T cells were transfected with FuGENE Transfection Reagent (Promega, E2312) according to the manufacturer's instructions.

### Immunofluorescence

HEK293T cells were seeded onto glass coverslips, washed with PBS, and fixed in pre-warmed 4% paraformaldehyde for 20 min at room temperature. After three PBS washes, cells were permeabilized with 0.2% Triton in PBS for 30 min and they were blocked with 1% BSA and 4% serum in PBS. The specific primary antibodies were incubated overnight at 4°C, and the secondary conjugated antibodies were incubated for 1.5 h at room temperature. The coverslips were mounted with Fluoromount-G with DAPI (4′, 6′-diamidino-2-phenylindole) (ThermoFisher Scientific, 00-4959-52).

### Proximity ligation assay (PLA)

HEK293T cells were seeded onto glass coverslips, washed with PBS, fixed in pre-warmed 4% paraformaldehyde for 20 min at room temperature, and permeabilized with ice-cold methanol at −20°C for 20 min. After 1 h of incubation with the blocking solution in a pre-heated humidity chamber at 37°C, cells were incubated overnight at 4°C with the specific primary antibodies. Afterwards, we performed the PLA assay according to the manufacturer's instructions (Duolink *In Situ*-Fluorescence, Sigma-Aldrich, DUO92008) and the coverslips were mounted with Duolink *In Situ* Mounting Medium with DAPI (Sigma-Aldrich, DUO82040).

### Western blotting and co-immunoprecipitation assays (Co-IP)

HEK293T cells were homogenized in lysis buffer (50 mM Tris HCl pH 7.4, 1.5 mM MgCl_2_, 5 mM EDTA, 1% Triton X-100, 50 mM NaF and 1 mM Na_2_VO_3_) containing a protease inhibitor cocktail (Complete Mini-Protease Inhibitor Cocktail, Roche, 11873580001). Homogenates were centrifuged at 13,200 rpm (FA-45-30-11 Rotor) for 15 min at 4°C and the protein concentration of the supernatant was quantified by the BCA method (ThermoFisher Scientific, 23225), resolved in sodium dodecyl sulfate-polyacrylamide gels (SDS-Page) and transferred onto PVDF Immobilon-P membranes (Merck, IPVH00010). The membranes were blocked with 5% defatted milk in TBS-T buffer (25 mM Tris, 50 mM NaCl, 2.5 mM KCl, 0.1% Tween-20). Afterward, the membranes were blotted with the specific primary antibodies, which were detected using secondary antibodies coupled to horseradish peroxidase. Proteins were processed for chemiluminescence with Amersham ECL Prime Western Blotting Detection Reagent (Cytiva, RPN2236) and visualized by iBrBand CL1000 Imaging System (ThermoFisher Scientific). Band intensity was measured using ImageJ software (NIH, http://rsb.info.nih.gov/ij).

For co-immunoprecipitation assays, 1 mg of total protein lysate was incubated with the specific antibody for 6–8 h at 4°C followed by incubation with Protein G Sepharose™ 4 Fast Flow (Cytiva, GE17-0618-01) overnight at 4°C. Beads were softly washed with lysis buffer, resuspended in Laemmli Buffer, heated at 95°C, and analyzed by SDS-Page and western blotting.

### Image acquisition and processing

Super-resolution images were acquired with a Leica TCS SP8 X White Light Laser confocal microscope with Hybrid spectral detectors and HyVolution (Leica Microsystems, Wetzlar, Germany) using the Leica LAS X software (version 3.1.5). Image deconvolution was performed with Huygens Essential software v.4.4 0p6 (SVI, Leiden, The Netherlands). Image processing and analysis were performed using the Leica Application Suite X (LAS-X) software (Leica Microsystems, Wetzlar, Germany) or ImageJ (NIH, http://rsb.info.nih.gov/ij).

#### Mitochondrial network morphology

To analyze mitochondrial network parameters, an ImageJ macro called Mito-Morphology was used (Dagda et al., 2009). First, a region of interest (ROI) was drawn around three representative areas of the cell. Second, a binary image was obtained, and the same threshold (Otsu) was applied. Finally, mitochondria of the selected part of the cell were measured using the Mito-Morphology macro. The average of circularity (how with closely the measured mitochondria represent a circle, being 1 a perfect circle) and the number of structures per ROI (fragmentation aspect) were determined.

#### Lysosome morphology

The analyses of lysosomes were assessed using ImageJ/Fiji (NIH). For LAMP-1 fluorescence intensity ROIs were drawn around representative areas of the cell. Three regions next to the cells without fluorescence were taken as background. The corrected total cell fluorescence (CTCF) formula was used: CTCF = integrated density – (area of selected cell × mean fluorescence of background readings). For lysosome area, maximum intensity projections were generated from Z-stacks followed by automated 8-bit Otsu-thresholding, and the binary images were evaluated to obtain the LAMP-1 total area in each cell.

### Statistics

Statistical analysis was performed using GraphPad Prism (version 8.0.1; GraphPad Software, La Jolla, CA, USA) with a minimum of three independent experiments. Normality was assessed by the Kolmogorov–Smirnov test. The specific test applied in each case is indicated in the figure legend. *P*-values less than 0.05 were considered significant. *P*-values are indicated by asterisks *P<0.05, **P<0.01, ***P<0.001.
